# Specific IgE response to purified and recombinant allergens in latex allergy

**DOI:** 10.1186/1476-7961-3-11

**Published:** 2005-08-10

**Authors:** Viswanath P Kurup, Gordon L Sussman, Hoong Y Yeang, Nancy Elms, Heimo Breiteneder, Siti AM Arif, Kevin J Kelly, Naveen K Bansal, Jordan N Fink

**Affiliations:** 1 Allergy-Immunology Division, Medical College of Wisconsin Milwaukee, WI, USA; 2 Research Service, V A Medical Center, Milwaukee, WI, USA; 3 University of Toronto, Ontario, Canada; 4 Biotechnology and Strategic Research Unit, Rubber Research Institute of Malaysia, Kuala Lumpur, Malaysia; 5 Department of Pathophysiology, Medical University of Vienna, Vienna, Austria; 6 Department of Mathematics, Marquette University, Milwaukee, WI, USA

## Abstract

**Background:**

In recent years, allergy to natural rubber latex has emerged as a major allergy among certain occupational groups and patients with underlying diseases. The sensitization and development of latex allergy has been attributed to exposure to products containing residual latex proteins. Although improved manufacturing procedures resulted in a considerable reduction of new cases, the potential risk for some patient groups is still great. In addition the prevalent cross-reactivity of latex proteins with other food allergens poses a major concern. A number of purified allergens and a few commercial kits are currently available, but no concerted effort was undertaken to evaluate them.

**Methods:**

We studied 11 purified latex allergens, Hev b 1 to Hev b 10, and Hev b 13 along with several crude allergen extracts and two commercial ImmunoCAP assays to evaluate specific IgE antibody in the sera from latex allergic patients and controls. Health care workers and spina bifida patients with clinical symptoms of latex allergy, spina bifida patients without latex allergy, and non-atopic health care workers have been studied.

**Results:**

The results suggest that Hev b 2, 5, 6, and 13 together identified over 80 percent health care workers with latex allergy, while Hev b 6 along with Hev b 1 or 3 detected specific IgE antibody in all sera studied from patients with spina bifida and latex allergy. The ImmunoCAP results using both Hev b 5 amplified and non-amplified closely agreed with the clinical diagnosis of latex allergy in health care workers and in spina bifida.

**Conclusion:**

Although the purified allergens and crude extracts reacted diversely with IgE from different patient groups, the results indicated that use of certain combinations of purified recombinant antigens will be useful in commercial kits or in in-house assays for detecting specific IgE antibody in the sera. The results suggest that a combination of Hev b 2, 3, 5, 6, and 13 together detected specific IgE in 80% of the sera from latex allergic patients. Both ImmunoCAPs correctly identified over 95% of latex allergic patients, however, showed reactivity with a few normal control subjects

## Background

During the 1980's and 90's, allergy to natural rubber latex had posed serious concerns, particularly in certain occupational groups exposed to latex allergens [1-4]. Among these occupational groups, health care workers (HCW) and patients with spina bifida (SB) constitute the two major populations exposed to various natural rubber latex products and have a high frequency of manifestations of latex allergy. Sensitization and the development of latex allergy have been attributed to the exposure to products containing residual latex proteins. Although considerable advances have been made in the diagnosis and patient care, no standardized tests or reagents are currently available that can be reliably and safely used in the diagnosis of latex allergy [3-5]. A crude latex extract from a clone of Malaysian rubber tree *Hevea brasiliensis*, clone RRIM 600 has been made available for evaluation and was proposed as a candidate allergen for skin test and in *in vitro *specific serum IgE assays [6,8]. This extract has been widely tested as a skin-testing reagent and has been evaluated by the multi-center latex skin testing study task force with success, although the clone is classified as an unstable phenotype with variability in the latex composition [9]. Crude extracts are not appropriate candidates as standardized antigens due to their variability, lack of dependability, irrelevant cross reactivity, and questionable safety in *in vivo *use such as skin testing. In recent years a number of genes encoding relevant antigens from natural rubber latex have been cloned and the proteins expressed [10]. However, only a few studies have been carried out to evaluate these conventionally purified or cloned and expressed allergens [5]. Currently, there are 13 *Hevea *latex allergens recognized by the IUIS Allergen Nomenclature Committee [10].

In recent years, several semi-automated *in vitro *assays have been developed commercially for detecting latex specific IgE antibody. In the present study, we investigated latex specific IgE in the sera of patients and controls using purified and crude latex allergens prepared from non-ammoniated Malaysian natural rubber latex extracts and glove extracts. The extracts were evaluated in an ELISA and the results compared with ImmunoCAP, a widely used semi-automated commercial assay for IgE antibody. The purified antigens reacted diversely with different patient sera by ELISA and no single allergen reacted with IgE from all proven latex allergic patients studied. However, Hev b 2, 5, 6, and 13 together and Hev b 6 with Hev b 1 or 3 demonstrated IgE from majority of HCW patients and spina bifida patients respectively. The ELISA results were comparable to ImmunoCAP, but the latter agreed more closely with clinical diagnosis.

## Methods

### Patients and controls

A total of 36 HCW were studied, of which 10 had no clinical symptoms of latex allergy; the remaining 26 subjects had clinically proven latex allergy [3,5]. Among the 21 SB patients studied, 13 had clinical latex allergy [11]. Latex allergy in health care workers was diagnosed by (a) a history of skin and respiratory symptoms often progressing from contact dermatitis through urticaria to asthma and anaphylaxis on latex contact, usually with latex glove powder inhalation, or (b) immediate wheal and flare skin reaction to latex glove antigens, (c) a history of reaction to cross-reactive latex antigens such as bananas or other fruits, and/or (d) serum IgE antibodies to latex glove extracts carried out by ELISA. Latex allergy in SB patients was diagnosed by a history of perioperative anaphylaxis and/or the demonstration of respiratory symptoms on latex glove powder contact, and/or the demonstration of antibody to latex antigens and a history of cross-reaction to food allergens [11]. All sera were evaluated for latex specific IgE antibody using a Malaysian non-ammoniated latex extract, two glove extracts routinely used in our laboratory to confirm the diagnosis [11,12].

### Latex Antigens

Four crude latex extracts and 11 purified and recombinant allergens from *H. brasiliensis *latex were used for *in vitro *studies of latex specific serum IgE antibody. The purified allergens used in the study are listed in Table 1. All antigens were used in an ELISA to evaluate latex specific IgE antibody in sera of patients with latex allergy and normal healthy controls [5,11,12]. Latex collected after tapping *H. brasiliensis *trees (rubber trees) was shipped frozen to the laboratory from Malaysia. The clear serum phase of the latex was collected after centrifugation of the coagulated latex as described previously [5,12,13]. This extract designated as Malaysian non-ammoniated latex (MNA) was characterized and used in ELISA as described previously [5]. Another crude latex extract was from clone RRIM 600 and was obtained from Greer Laboratory [7]. Two latex glove extracts were also used in the study. These gloves were selected from two different manufacturing sources, one with more extractable protein, while the other one with a lower latex protein content. The allergens were extracted from pieces of latex gloves by stirring with PBS in a flask for 15 min at room temperature as previously described [14,15]. We used two different ImmunoCAPs; in one the crude latex was used to make the CAPs, while in the other in addition to the regular CAP, Hev b 5 was also supplemented. This modification was devised to remedy the lack of Hev b 5 in the clotted serum of rubber latex.

**Table 1:** Purified Latex Allergens and their Characteristics

**Allergen**	**Biochemical Function**	**Alternate Name**	**Molecular Size kDa**	**Significance in the Diagnosis**
Hev b 1	Biosynthesis of polyisoprene	Rubber elongation factor	14.9	SB
Hev b 2	Beta 1-3-glucanase defense protein	-	35.1	HCW
Hev b 3	Biosynthesis of polyisoprene	-	22.3	SB
Hev b 4	Micro helix protein	-	50–57	-
Hev b 5	Structural protein		16	HCW
Hev b 6	Plant defense	Prohevein	20	SB/HCW
Hev b 7	Esterase inhibitor of polyisoprene	Patalin	42.9	-
Hev b 8	Profilin	Profilin	13.9	-
Hev b 9	Enolase		47.7	-
Hev b 10	Manganese superoxide dismutase	-	22.9	-
Hev b 13	Esterase	-	43	HCW

Three of the allergens Hev b 2, Hev b 4, and Hev b 13 were purified from latex by the Malaysian laboratory (HYY) as previously described [5,16,17]. The genes for Hev b 1, 3, 5, 6, 7, 8, 9, and 10 were cloned from cDNA libraries and Hev b 1, 3, 5, and 6 were expressed in the Medical College of Wisconsin laboratory (VPK), while Hev b 7, 8, 9, and 10 were cloned and expressed in the University of Vienna laboratory (HB) [18,27].

### Characterization of latex antigens

The protein profile of the extract was studied by sodium dodecyl sulfate polyacrylamide gel electrophoresis. Electrophoresis was carried out by loading 10 micrograms of proteins on a 12% SDS polyacrylamide mini gel and running at 200 mv/cm for 40 to 50 minutes [5]. The gels were stained with Coomassie brilliant blue R-250 and the stained bands in the gel were compared and the molecular sizes ascertained. The reactivity of antigens to serum IgE was studied using pooled sera from HCW and SB patients with latex allergy and controls by ELISA and Western blot.

### Latex specific IgE by ELISA

The MNA, Clone RRIM 600, glove extract antigens, and purified latex proteins were coated at a concentration of 5-μg protein/ml. All dilutions and coating concentrations of the antigens and reagents were derived from checkerboard titration using latex positive and negative sera. The ELISA was performed as previously described [5]. Briefly, one hundred micro liters of the preparations were added to the wells of polystyrene micro titer plates (Immunolon II HB, Therma Lab Systems, Franklin, MA). The plates were incubated at room temperature for 3 hours, followed by a further incubation at 4°C overnight. After washing the plates with PBS, containing 0.05% Tween 20 (PBS-T), the wells were blocked with 0.5% BSA in PBS-T. The wells were again washed and 100-μl of 1:25 dilution of the serum added to each well, incubated at room temperature for 3 hours, and washed as before. One hundred-μl of biotinylated mouse, anti-human IgE monoclonal antibody (Zymed Laboratories, Inc., San Francisco, CA) was added to each well and the plates were incubated for 1 hour at room temperature, washed as before and 100 μl of 1:2000 dilution of streptavidin peroxidase was added to the wells. This was followed by incubation for 30 minutes and washing again. Finally, the peroxidase activity was developed with o-phenylenediamine substrate in citrate buffer. The color was developed for 15 minutes in a dark chamber and the reaction stopped by the addition of 25 μl of 2N H_2_SO_4 _solution. The color was read in an ELISA plate reader using a 490 nm filter (Molecular Devices; Sterling, VA). The optical density (O.D) values were corrected by subtracting the blank values and the average of three wells was taken. A value exceeding mean plus two standard deviation (SD) of HCW and SB patients without latex allergy was taken as a cut off value for positivity.

### ImmunoCAP

The Pharmacia ImmunoCAP was used to demonstrate latex specific IgE in the sera of patients and controls according to the instructions of the manufacturer. Both Hev b 5 amplified (rk82) and non-amplified (k82) ImmunoCAPs were used. The protocol of the manufacturer was followed, and a value of 0.35 kU_A_/L or more was considered positive.

### Statistical analysis

The mean O.D values for all allergens were calculated and the results were analyzed by the multivariate analysis of variance (MANOVA). A P-value of 0.05 was considered significant. When a significant difference was detected, a stepwise discriminant analysis was also performed to select the significant allergens that delineate the positive and negative groups by their reactivity or non-reactivity with IgE by ELISA. The variables with P-value of < 0.05 were chosen as the significant variables, while the variables with P-value > 0.20 were removed at each step of the discriminant analysis. Using all the significant allergens, Fisher discriminant functions for the latex allergic and non-allergic HCW and SB subjects were calculated. Based on this, each case was assigned two Fisher discriminant scores, one for the latex allergic group and the other one for the non-allergic group. Each subject was classified into a group (latex allergic or non-allergic) based on the higher corresponding Fisher discriminant score value as the case may be [5,28].

## Results

### Characteristics of the antigens

**Figure 1:**
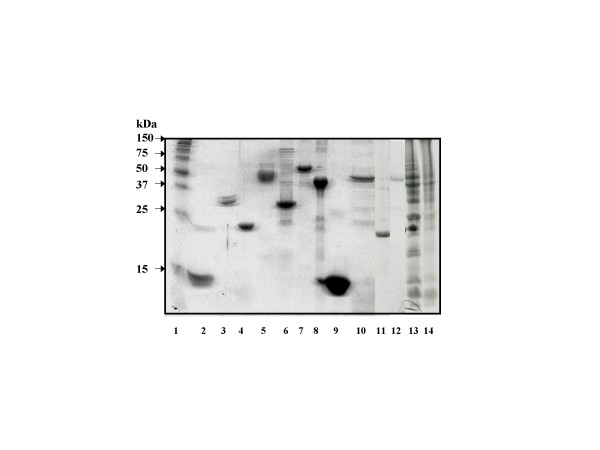
Sodium dodecyl sulfate polyacrylamide gel electrophoresis profile of latex allergens. Allergens (10 μg) were subjected to electrophoresis in 12% SDS-Gel and stained with Coomassie brilliant blue. 1 – Molecular weight standards; 2–12 – Hev b 1 to 10 and Hev b 13; 13 – Crude latex (MNA); 14 – RRIM Clone 600 latex.

The protein profiles using sodium dodecyl sulfate polyacrylamide gel electrophoresis (SDS-PAGE) of MNA, Clone RRIM600 and the various purified latex antigens are shown in Figure 1. MNA and Clone RRIM600 showed a number of bands in SDS-PAGE, while most of the purified allergens used in the study showed single bands. A few of the purified antigens showed additional weak bands in the gel, the immunoblots of the protein reacted with IgE from latex allergic patients showing IgE binding with only the major bands and not with the weak bands. The two glove extracts showed very weak bands. The Western blots of both glove extracts, MNA, and Clone RRIM600 showed multiple reactive bands with the pooled latex allergic patient sera, but not with the normal control sera.

### Latex specific IgE in the sera

**Figure 2:**
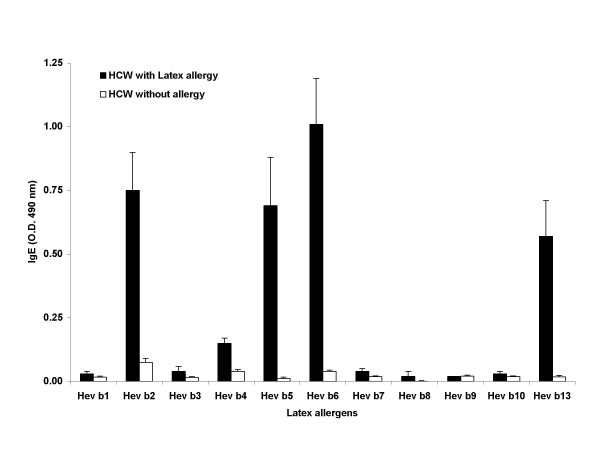
Anti-latex IgE antibodies in the sera of HCW patients. IgE antibody against various purified latex allergens in the sera of health care workers with latex allergy and those with no clinical symptoms of latex allergy were studied by ELISA.

**Figure 3:**
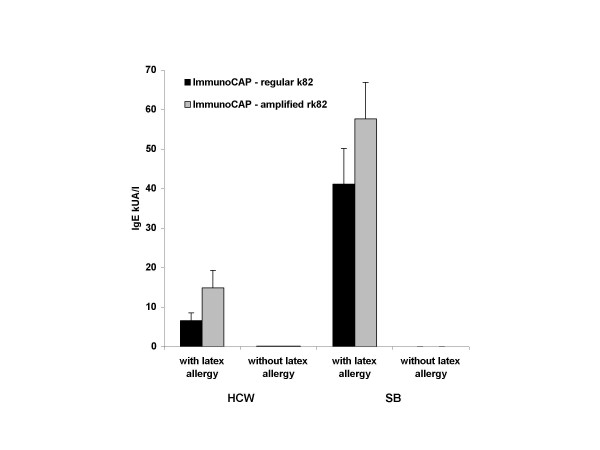
Latex specific IgE antibody in the sera of spina bifida (SB) patients and health care workers. Health care workers (HCW) and spina bifida (SB) patients with and without latex allergy were studied for specific IgE by ImmunoCAPs, regular (k82) and ImmunoCAPs amplified with Hev b 5 (rk82).

Of the 36 HCW evaluated, 26 were symptomatic with urticaria or asthma on latex allergen exposure; 10 subjects had no symptoms on exposure to latex products in the health care workplace. All 36 subjects included in this study were exposed to latex proteins present in gloves or other latex products. The IgE reactivity of the sera from HCW patients to11 purified latex antigens by ELISA is shown in Figure 2. Hev b 2, 4, 5, 6, and 13 showed significant binding to IgE in the patients' sera, while Hev b 1, 3, 7, 8, 9, and 10 failed to show significant binding to IgE compared to controls (Fig. 2). In ImmunoCAP, 25/26 HCW patients showed 0.35 kU_A_/L or more, while one normal subject without latex allergy was also positive (Fig. 3). One out of the 26 patients who failed to show latex specific IgE by the ImmunoCAP showed strong reactivity with Hev b 5 by ELISA and Hev b 5 amplified ImmunoCAP. On the other hand, one patient negative to all crude antigens and most purified antigens showed strong reactivity to Hev b 5 and amplified Hev b 5 ImmunoCAPs. This patient also showed significant IgE to Hev b 5 and Hev b 13 by ELISA. The solitary HCW patient that showed strong reactivity with Hev b 1 and Hev b 3 also reacted with most other latex allergens studied. Hev b 7, 8, 9, and 10 invariably showed only very weak reactions with specific IgE in the sera of most patients tested, while Hev b 2, Hev b 6, and Hev b 13 consistently showed high levels of IgE in more patients compared to other purified allergens. Three out of 10 normal subjects also showed IgE to Hev b 2 in their sera, but the levels were comparatively lower than those detected in patients. Hev b 13 also failed to react with two latex allergic patients, but did not show any reactivity with the normal controls.

**Figure 4:**
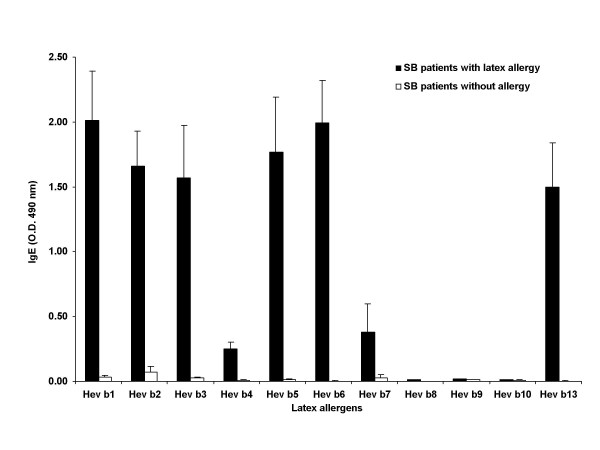
IgE antibody against various purified latex allergens in SB patients. The sera of spina bifida patients with latex allergy and those without clinical symptoms of latex allergy were studied for the presence of latex specific IgE using recombinant latex allergens by ELISA.

The reactivity of various allergens to the IgE of SB patients are shown in Figure 4. All 13 latex allergic patients showed significantly elevated IgE levels by both ImmunoCAPs and ELISA using crude latex extracts, while none of the non-allergic SB showed any reactivity (Fig. 3). Both Hev b 1 and Hev b 3 demonstrated strong reactivity with IgE of 11/13 patients; the remaining two patients had low levels of IgE against these two allergens. Hev b 6 demonstrated strong IgE binding to all but one SB patient with latex allergy, but had no reactivity with SB patients without latex allergy. Three patients each failed to react with Hev b 5 and Hev b 13, while only three showed binding of IgE to Hev b 7. Hev b 8, 9 and 10 failed to show binding with any of the patients or control sera. None of the SB patients without latex allergy showed any significant reactivity with IgE to any of the latex allergens except Hev b 2, which showed strong reactivity with only one patient.

**Figure 5:**
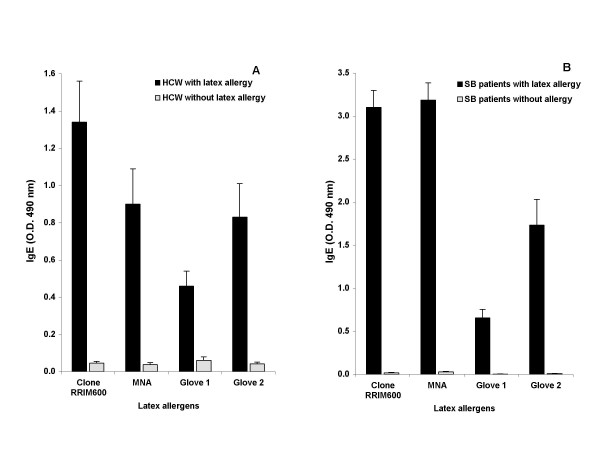
IgE reactivity of HCW and SB patients to latex and glove extract antigens. Latex antigens from *Hevea brasiliensis*, Clone RRIM 600 and extracts from two examination gloves were studied for IgE binding using sera from spina bifida and health care workers suing ELISA.

The reactivity of HCW and SB sera against the four crude antigens are shown in Figure 5A and 5B. Both NRL extracts reacted strongly with both SB and HCW, with Clone RRIM600 showing more reactivity with HCW patients. Among the crude extracts studied, Glove 1 invariably showed less reactivity. All four antigens showed reactivity with the sera from a few control subjects.

The binding of IgE to allergens Hev b 1 to Hev b 6 and Hev b 13 showed a significant difference (P < 0.05) between SB patients with and without latex allergy when studied individually. Hev b 7 to Hev b 10 failed to show any significant IgE binding reactivity between these two groups. Among the HCW patients with latex allergy studied, strong reactivity was detected only with Hev b 2, Hev b 5, Hev b 6, and Hev b 13. When all 11 purified latex proteins were used together and analyzed the data by MANOVA, a significant difference was detected with latex allergic and non-allergic subjects from both HCW and SB groups (P < 0.05).

Stepwise discriminant analysis of the ELISA data from SB patients selected Hev b 1, Hev b 3, and Hev b 6 and together these antigens delineated all the latex allergic and non-allergic subjects. The Fisher discrimination function for the positive group is -10.549 + 11.569 Hev b 1 -9.189 Hev b 3 + 5.443 Hev b 6, and for the negative group is -0.694 + 0.098 Hev b 1 -0.068 Hev b 3 + 0.027 Hev b 6. All the SB subjects studied could be classified into latex allergic or non-allergic, and the specificity and sensitivity were found to be 100% by ELISA using these allergens.

The Stepwise discriminant analysis on HCW workers selected only Hev b 6 as the major allergen, perhaps due to the fact that this protein is the major allergen with marked specificity. The Fisher discriminant function for the latex allergic patient group when Hev b 6 alone is used is -1.511 -1.627 Hev b 6, and for the non-allergic group is -0.694 + 0.063 Hev b 6. This analysis correctly classified 17 out of 26 patients as having latex allergy and all 10 HCW subjects without symptoms. However, Hev b 2, Hev b 5, and Hev b 13 also showed significant reactivity and hence, discriminant analysis of the IgE binding of Hev b 2, Hev b 5, Hev b 6, and Hev b 13 was also carried out. The sensitivity and specificity using only Hev b 6 or using Hev b 2, Hev b 5, Hev b 6, and Hev b 13, and using all allergens Hev b 1 to Hev b 13 were carried out for all patients and the results indicate a sensitivity of 65 to 85% and a specificity of 100% (Table 2).

**Table 2:** Sensitivity and specificity of purified latex allergens in the diagnosis of latex allergy in health care workers (HCW)

**Latex Allergens**	**Specificity**	**Sensitivity**	**Overall Correct Agreement**
Hev b 6	100%	65.40%	75%
Hev b 2, 5, 6, & 13	100%	73.1%	80.6%
All allergens Hev b 1 to Hev b 13	100%	84.6%	88.9%

## Discussion

The results indicate that crude NRL allergens including an extract from Clone RRIM600 demonstrate strong reactivity with IgE from latex allergic HCW patients. Both glove extracts, in spite of their differences in protein content and failure to show distinct bands in SDS-PAGE, demonstrated similar reactivity as shown by MNA and Clone RRIM600 with both groups of patients. The single patient negative by unamplified ImmunoCAP reacted strongly to the amplified ImmunoCAP with Hev b 5 indicating that Hev b 5 is important for the diagnosis of some of the HCW patients with latex allergy. None of the other purified latex allergens studied reacted with IgE from this patient. Our results indicate that Hev b 1, 3, 4, and 7 through 10 have little or no value in the demonstration of IgE in HCW patients with latex allergy. In a previous study, we have shown that Hev b 2, 6, and 7 were useful in demonstrating IgE in the sera of HCW patients with latex allergy [5]. Since we did not test Hev b 5 and 13 in the previous study, the present results indicate a more complete representation of all the relevant latex allergens and their reactivity with the sera from different groups of patients and controls. The results of the present study suggest the usefulness of Hev b 2, 5, 6, and 13 together in the diagnosis of latex allergy in HCW.

SB patients with latex allergy showed antibody responses to a different set of latex allergens. Both ELISA and ImmunoCAP showed strong agreement in demonstrating latex specific IgE in the sera of most of these patients. The findings indicate that a combination of Hev b 6 and Hev b 1 or 3 would demonstrate specific IgE in the sera of all patients with SB and latex allergy.

Although crude latex antigens are efficient in demonstrating IgE antibodies in latex allergic patients, such extracts are not appropriate as standardizable allergen reagents due to the inherent variability, complexity of allergenic components, and in the presence of cross reactive allergens. The present study suggests that by selecting significant antigens and by reconstituting known amounts of purified allergens, it may be possible to obtain standardizable preparations to demonstrate IgE antibody in the sera of HCW and SB patients with latex allergy. From the present study and from previous multi-center studies, it has been shown that the presence of latex specific IgE in HCW patients' sera can be demonstrated using a mixture of Hev b 2, 5, 6, and 13 and in SB patients with latex allergy by the use of Hev b 6 along with Hev b 1 or Hev b 3. It is not possible to derive a cut-off value for delineating the allergic patients from normal controls due to the high variability in the responses of the patients. However, additional patients may be studied before finally selecting the allergens and their proportions in the mixture for a more standardizable reagent and for devising a delineation titer.

The present study suggests the need to develop more specific reliable and reproducible allergen preparations for *in vitro *detection of latex allergen specific IgE. Kim and coworkers demonstrated that specific IgE levels to latex allergens in the sera of patients were symptom dependent and that patients with asthma showed higher levels of specific IgE compared to those with dermatitis alone [29,30]. Although other investigators demonstrated false positives and false negative reactions with ImmunoCAP, Alastat and HY-TEC methods, in the present study our results were more clear -cut with less false positive and false negative reactions [7,8]. In the present study, we have observed a more stronger reactivity with patient serum by Hev b 5 complemented ImmunoCAP compared to regular ImmunoCAP. However, the Hev b 5 amplified CAPs also showed more reactivity with normal control subjects without latex allergy. Moreover, the reactivities of HCW and SB patients' serum IgE with the purified antigens were more consistent than with crude latex extracts and no false reactivity was detected. Taken together, the present results indicate that ImmunoCAP system using purified relevant allergens, could be more dependable and reliable in *in vitro *demonstration of latex allergen specific IgE in the sera of latex allergic patients. The results suggest that a combination of Hev b 2, 3, 5, 6, and 13 would demonstrate IgE antibody in the majority of latex allergic patients.

## Conclusion

The results indicate that ImmunoCAP, particularly amplified with Hev b 5, was useful in demonstrating specific IgE in the sera of latex allergic patients. When all purified latex allergens were used together in ELISA, about 89% of patients with latex allergy were correctly identified. We conclude from these results that selection of significant recombinant allergens and reconstitution of these purified antigens in immunoassays, such as ELISA, will provide standardizable reagents for demonstrating specific IgE in the sera of patients with latex allergy. These selected purified allergens can be used for more reliable results in automated assays such as ImmunoCAP.

## Competing interests

The author(s) declare that they have no competing interests.

## Authors' contributions

VPK, GLS, JNF, and KJK designed the study. VPK and NE conducted the immunoassays. HYY, HB, SAMA, and VPK provided the recombinant allergens. GLS, JNF, and KJK provided sera. NKB planned the experiment and analyzed the data. All authors contributed towards the manuscript preparation. VPK coordinated the study.
